# Considering health literacy in cardiovascular disease management: a qualitative study on healthcare professionals’ and patients’ perspectives

**DOI:** 10.1186/s12913-022-08455-8

**Published:** 2022-09-05

**Authors:** Adèle Perrin, Eléonore Damiolini, Anne-Marie Schott, Jéremy Zermati, Estelle Bravant, François Delahaye, Alexandra L. Dima, Julie Haesebaert

**Affiliations:** 1 Research on Healthcare Performance (RESHAPE), INSERM U1290, Université Claude Bernard Lyon 1, Lyon, France; 2 Hospices Civils de Lyon, Pôle Santé Publique, Service Recherche et Epidémiologie Cliniques, F-69003 Lyon, France; 3 Hospices Civils de Lyon, Hôpital Louis Pradel, Service de cardiologie, F-69500 Lyon, France

**Keywords:** Health literacy, Communication, Tailored strategy, Implementation, Qualitative study

## Abstract

**Background:**

Implementing practices adapted to patient health literacy (HL) is a promising avenue for improving their outcomes in the context of cardiovascular diseases (CVD). The health communication skills of healthcare professionals (HCPs) and the quality of information provided are essential for low-HL patients. We aimed to explore HCP knowledge about HL, patients’ and HCPs’ views on current practices regarding low-HL patients, and facilitators and barriers to adapting communication to patients’ HL level, in order to prepare the implementation of a complex intervention dedicated to improve CVD management for low-HL patients.

**Methods:**

We conducted face-to-face semi-structured interviews with HCPs practicing in cardiology units and patients hospitalized for CVD. The study design and analysis were based on the Theory of Planned Behavior for HCPs and on the framework of Health Literacy and Health Action for patients. Deductive and inductive thematic analysis were used. Barriers and facilitators were structured into an Ishikawa fishbone diagram and implementation strategies were selected to address resulting themes from the Expert Recommendations for Implementing Change (ERIC).

**Results:**

Fifteen patients and 14 HCPs were interviewed. HCPs had partial knowledge of HL dimensions. Perceptions of HCPs and patients were not congruent regarding HCP-patient interactions and information provided by hospital and community HCPs. HCPs perceived they lacked validated tools and skills, and declared they adapted spontaneously their communication when interacting with low-HL patients. Patients expressed unmet needs regarding communication during hospital discharge and at return to home.

**Conclusion:**

To implement HL-tailored practices in this setting, our results suggest that several implementation strategies will be valuable at individual (engaging patients and their family), interactional (educating and training of HCPs about HL), and organizational levels (creating a multidisciplinary HCP interest group dedicated to HL).

**Trial registration:**

ClinicalTrials.gov, (NCT number) NCT03949309, May 10, 2019.

**Supplementary Information:**

The online version contains supplementary material available at 10.1186/s12913-022-08455-8.

## Background

Implementing practices adapted to each patient health literacy (HL) level constitutes a major challenge but improves their outcomes in the context of cardiovascular diseases (CVD), as recently emphasized by the American Heart Association [1]. HL is defined as *“people’s knowledge, motivation and competences to access, understand, appraise, and apply health information”* [2]. Understanding and implementing appropriate health behaviors regarding lifestyle and adherence to medication may be particularly difficult for low-HL CVD patients [3, 4] who are at high risk of medication errors following hospital discharge. In this context, HL acts as a mediator of other health determinants [5]. Indeed, low-HL patients face greater obstacles to access healthcare, communicate with providers, and engage in their care [6]. In discharge planning, specific attention to patients enables the promotion of a safe and effective transition in care. Boyle et al., have shown that low HL level is associated with worse post-discharge outcomes, including higher risks of adverse outcomes, such as hospital readmissions [7]. Thus, implementing practices [8] and a culture sensitive to HL in health services may be beneficial to patients as it would improve patient self-care and health outcomes [9]. Many strategies addressing HL issues have been developed and proven effective to improve access to care by addressing HL issues [10], however their implementation in daily practice remains suboptimal [1, 11].

Healthcare professionals (HCPs) play a key role in reducing HL-related barriers to healthcare by adapting their communication to foster patient knowledge, understanding, and engagement. However, the ability of HCPs to assess patient HL level and needs is often poor [12, 13]. HL screening is not yet implemented in daily clinical practice, partly because of the lack of awareness and training of HCPs regarding HL, but also because of the lack of validated tools that can be used within the time constraints of daily practice [14], and most HCPs overestimate patients’ HL level [15–19]. They usually consider patients’ language skill, educational level, or social status as indicators of HL, although these features do not always constitute good proxies of HL and can be perceived as stigmatizing [20]. In addition, access to multiple and conflicting information, as well as materials that are often very difficult to understand and require simplification, add up to the difficulties faced by low-HL patients [21]. In this context, both HCP communication skills and the quality of the written and oral information provided are essential for low-HL patients [12]. Thus, raising the awareness of HCPs regarding HL definition and consequences on health, and providing them with methods and tools to assess patient HL level, may help them to implement HL-sensitive practices and to systematically integrate HL in CVD prevention and management [22]. The ‘universal precautions toolkit’ has been promoted by the Agency of Health Research and Quality (AHRQ) to improve communication and HL implementation in CVD health care [1] and to help professionals change their practices.

The present study is part of a stepwise approach aiming at developing and implementing a HL-tailored intervention for the improvement of HCP skills to deliver clear and actionable information to low-HL CVD patients during the transition from hospital to home.

Getting a deeper understanding of the modifiable determinants related to HCP communication practices and of the barriers and facilitators required for HL-tailored practice implementation is a prerequisite to identify behavior change techniques to target relevant determinants in the development of the CVD management intervention, and to select appropriate implementation strategies for this future intervention.

The primary objective of this qualitative study was to explore HCP knowledge about HL, including the way they define HL and appraise patient HL level in their daily practice. The secondary objectives were, to explore how HCPs tailor their communication when interacting with low-HL patients and to compare their perceptions with the needs expressed by patients. Finally, we aimed to identify modifiable determinants (barriers and facilitators) at both HCP and patient level for implementing interventions to improve low-HL patient-HCP interactions regarding the management of CVD.

## Methods

### Aim, design, and setting of the study

We conducted a qualitative cross-sectional study based on face-to-face semi-structured interviews with HCPs practicing in cardiology units of the *Hospices Civils de Lyon,* and with patients hospitalized in cardiology units of the *Hôpital Louis Pradel* (*Hospices Civils de Lyon*) for either myocardial infarction (MI) or heart failure (HF). Our study and the reported results were guided by the consolidated criteria for reporting qualitative research [23].

The study was underpinned in its design and analysis by two theoretical models: the Theory of planned behavior (TBP, [24]) for HCPs and the framework of Health Literacy and Health Action (HLHA, [25]) for patients. Using the TBP constructs, we inquired about HCP behavioral intention to adapt their communication according to patient HL level and determinants (norms, perception of control, attitude). Similarly, following the HLHA framework, we explored with patients the possible motivational and volitional determinants through which HL may affect health action dimensions such as patient-provider interaction. We inquired about their needs 1 month after hospital discharge.

HCPs were asked about their representation and definition of HL, their attitudes regarding HL, and their perceptions of control over adapting communication to patients’ HL difficulties. Patients were invited to discuss their knowledge and difficulties in managing their disease, their perceived self-efficacy, their experiences after hospitalization (motivational, volitional, or system determinants), and how they accessed health information. The interview guides are presented in Additional file 1: Table A and B, Appendix 2.

To facilitate the identification of barriers and facilitators for implementing HL-tailored strategies about the management of CVD, we constructed a cause-and-effect diagram following the processes of gathering and organizing the potential causes for an effect [26].

### Characteristics of participants

We adopted a purposive maximum-variation approach to sample participants who represented different profiles of patients and HCPs [27].

A sample of HCPs from several professions involved in HF or AMI care was recruited: medical doctors, residents, assistant nurses, and nurses, contacted by e-mail directly or via word of mouth. The recruitment was made thanks to the collaboration of our research team with cardiologists of the hospital (one of them is a researcher affiliated in our laboratory) and thanks to professionals practicing in the services where the first quantitative phase of the study took place. An appointment was made with the HCPs interested in participating, during which an information letter was given to them, and oral consent was obtained before beginning the interview.

Patients were recruited within a larger mixed-method cross-sectional study named P-ILIADE [28]. The P-ILIADE study aimed to assess the prevalence of low-HL among the study population and to explore factors associated with HL levels. All patients included in the quantitative cross-sectional survey of the P-ILIADE study during their hospitalization were invited to participate in the qualitative part of the study (i.e. the present study). We interviewed patients 30 days after hospital discharge about their experiences and how they felt about the transition and early post-hospitalization period after the acute CVD episode. We aimed to interview participants with distinct characteristics in terms of disease (HF vs AMI) and HL level (adequate vs low). The latter was measured using the Brief Health Literacy Screening [BHLS, [29]] questionnaire validated in French hospital settings [30] within the context of the P-ILIADE study. The BHLS questionnaire was chosen for its short assignment duration and easy calculation of HL level, as it was necessary to be able to evaluate all patients of the P-ILIADE study. Hence, it was administered to all participating patients. Information letter was given at the inclusion in P-ILIADE study, oral consent for participating in the interview was obtained before the interview.

### Data collection

A PhD candidate in public health (AP), trained in interviewing and mentored by a health psychology researcher (ALD), recruited all the participants and conducted the interviews. Face-to-face interviews were carried out most of the time in a quiet hospital consultation room or, if necessary, in a waiting room. All participants were informed before the interview that it would be audio recorded.

We estimated that data saturation (*i.e.* participants expressing similar views and experiences on the topics discussed despite probing for diverse opinions would likely be reached after approximately 15 interviews per group of participants and this was the case for patients [31]; for HCPs, saturation was reached with 14 interviews. HCPs completed a brief demographic questionnaire regarding both individual (gender, age) and professional (profession, duration and place of exercise) information. Socio-demographic patient data (gender, age, disease, nationality) were retrieved from the P-ILIADE database.

### Data analysis

All interviews were audio recorded and transcribed verbatim. The transcriptions were analyzed independently by two researchers (AP and ED) using the NVivo qualitative data analysis software (QSR International Pty Ltd. Version 12, 2018). Quotes were identified according to gender (M [men] or W [women], HL level (L [Low] or A [Adequate]), patient (PA) or HCPs (HP), and inclusion number. We conducted a content analysis [32] combining deductive and inductive coding. The two theoretical models mentioned above used to develop the interview guides were also used to establish a priori the codebook for the analysis (deductive). The elimination or addition of certain themes allowed to adjust the codebook during the analysis (inductive). The two models were combined to represent the links between patient and HCP determinants of actions and interactions with the common aim of successful transition, longer-term management, and improved health outcomes (Additional file 1: Appendix 1). A triangulation of analyses was carried out between the AP, ED, and JH. Finally, we structured the identified barriers and facilitators using the “Ishikawa” Fishbone diagram [33]. The fishbone diagram is a technique used to explore potential sources of improvement of quality management process and construct interventions that guide to reorganization. We have used it here to summarize factors contributing to HL-tailored interactions and then link them to implementation strategies as proposed by the expert recommendations for implementing change (ERIC) recommendations [34]. ERIC is a compilation of implementation strategies employed to facilitate the use of evidence-based programs in healthcare contexts.

**Fig. 1:**
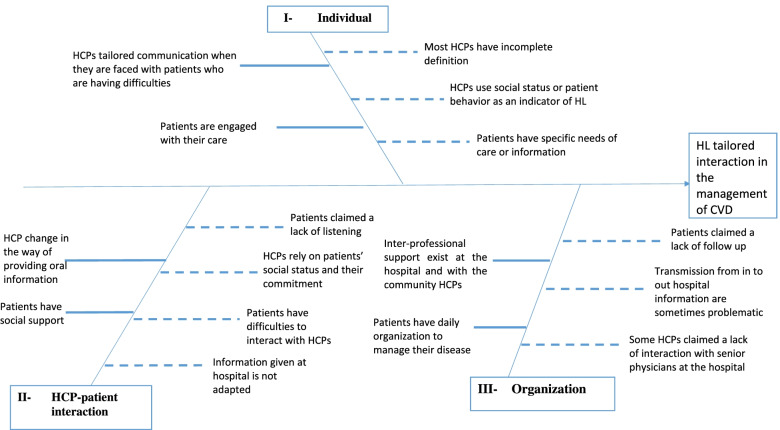
Fishbone diagram of both patients and HCPs-reported barriers and facilitators. A bold arrow on the left represents facilitators and barriers are on the right represented by dotted lines; illustrating levers to better interaction during the transition from hospital to home

We grouped the identified barriers and facilitators involved in the management of CVD for low-HL patients using the “Ishikawa” Fishbone diagram [33] (Fig. 1) into three levels: individual level, HCP-patient interaction level, and organizational level (Additional file 1: Appendix 3). Based on this structure, and guided by the HL universal precaution (AHRQ [35]) and the ERIC [34], we proposed implementation strategies.

## Results

### Sample characteristics

We interviewed 15 patients and 14 HCPs (Table 1 and Table 2): 5 medical doctors/residents, 6 nurses, and 3 assistant nurses. Among the 14 HCPs interviewed, 9 (64%) were women, 10 (71%) were aged under 50 years, and the mean (range) duration of interviews was 42 (24–57) minutes. Among the 15 patients interviewed, 10 (67%) had HF and 5 (33%) had an MI history, 8 (53%) were men, 9 (60%) had a low HL level, and their mean (range) age was 63 (39–84) years (Table 1). The mean (range) duration of interviews was 30 (min 18 – max 37) minutes.

**Table 1:** Patients’ characteristics

Identifier code	Disease	Age (years)	Sex	BHLS score (≤9, Low)	Nationality
LWPA1	HF	> 80	Woman	5	Other than French
LMPA2	MI	30–39	Man	9	French
LMPA5	MI	70–80	Man	4	French
LWPA7	MI	40–49	Woman	9	French
LWPA8	HF	50–59	Woman	7	French
LMPA9	HF	40–49	Man	4	French
LMPA11	HF	60–70	Man	3	Other than French
LWPA12	HF	> 80	Woman	8	French
LWPA13	HF	40–49	Woman	8	French
AMPA3	MI	40–49	Man	14	French
AMPA4	MI	60–70	Man	15	French
AMPA6	HF	60–70	Man	10	French
AMPA10	HF	60–70	Man	15	French
AWPA14	HF	70–80	Woman	12	French
AWPA15	HF	> 80	Woman	12	French

*Abbreviations*: *HF* Heart failure, *MI* Myocardial infraction, *W* Woman; *M* Man, *PA* Patient, *BHLS* Brief health literacy screening

**Table 2:** HCPs’ characteristics

Identifier code	Sex	Age group (in years)	Profession	Experience in the profession (in years)
MHP1	Man	50–59	Medical doctor	20
MHP2	Man	60–69	Medical doctor	39
WHP3	Woman	50–59	Nurse	23
WHP4	Woman	30–39	Nurse	10
WHP5	Woman	40–49	Nurse	2
MHP6	Man	30–39	Medical doctor	2
WHP7	Woman	50–59	Nurse	32
MHP8	Man	30–39	Medical doctor	4
WHP9	Woman	30–39	Nurse	10
WHP10	Woman	30–39	Assistant nurse	10
WHP11	Woman	20–29	Resident	4
WHP12	Woman	30–39	Assistant nurse	19
WHP13	Woman	30–39	Assistant nurse	20
MHP14	Man	40–49	Nurse	27

*Abbreviations*: *M* Man, *W* Woman, *HP* Healthcare professional

#### Healthcare professionals’ knowledge regarding health literacy

Among HCPs, the level of knowledge about HL was heterogeneous, as 5/14 HCPs were not able to provide a definition of HL. Other HCPs gave partial definitions that suggested links to one or more of Nutbeam et al.’s [36] dimensions of HL (functional, interactive, and critical). Most HCPs defined HL through its interactive dimension *e.g*. skills that can be used to extract information and applied to different circumstances and forms of communication.

*“It is the ability of a patient or a healthy person to integrate, understand, and implement actions in order to get better.” WHP13*

Finally, others described HL through its critical dimension i.e. skills that allow patients to critically analyze information and use it to exert a greater control on their life.

*“Literacy goes way beyond simple health literacy [you have to know what to shop for, how to read on food elements the salt composition...] you still have to know how to read, search for the information, and know what to do with it...” MHP2*

The only HCPs who provided an extensive integrative definition of HL were a nurse who was trained in therapeutic patient education and studied HL in this context, and her colleagues with whom she had shared her knowledge about HL.

“*it is the capacity of a person to first understand, to be able to read and to put into practice the things that we are going to be able to provide, the advice and recommendations related to health... and also how one can search for information, whether one is able to do so or not... ” WHP3*

The HCP representations of low-HL patients were often associated with patients’ social characteristics (e.g. older age, social deprivation) and current psychological state.

*“It’s not only about elderly patients, it’s also about patients who are in great social precariousness, who do not have all the means [...] there are depressed [...]. And these patients are among the people with low literacy skills.” WHP13*

Sometimes the patients’ ethnic background was considered by HCPs to be a proxy of their HL level.

*"There is another population there... I'm going to talk about the Mediterranean people, Spain, Italy, and Portugal, but we also have a lot of North African patients who are alone here, and these North African patients have very big problems of understanding and putting into practice...." MHP1*

Patients’ limited communication skills and reluctant attitudes regarding patient-provider interactions were also associated with low HL level.

*“he doesn't communicate, he doesn't answer questions, or he may be aggressive and always in denial. [...] Some people don't want to talk. A low level of literacy?“WHP13*

HCPs also estimated the HL level of their patients according to their behaviors or attitudes.

*“It can be precisely in relation to a question that the person has already asked me 4 times, and to which I have already answered 4 times, so I say to myself that either the question is not the right one that he wants to ask me, or the answer is not the right one that he wants to hear.” WHP3*

#### HCP-patient interactions: current practice perceived by HCPs and patients

##### HCP perspective

HCPs reported they dealt with low-HL patients by adapting their behaviors when recognizing difficulties in patients. Most interviewed HCPs (12/14) declared that they changed their behaviors when communicating with patients for whom the difficulties are apparent.

They stated that they provided patients with simplified explanations, using layman’s language and/or illustrations.

*“Indeed, we’re going to use simpler terms it's obvious... and then uh... we're going to ... so I'm simplifying... [...] or I'm going to explain to them what this term means, in a visual way, I try to put into images what I’m explaining. I sometimes have, we have teaching aids, we have a little plastic heart.” WHP5*

They also mentioned the importance of time: taking the time to let the patient ask questions and above all, taking the time to re-explain.

*“Well, we take maybe more time with him [...] to explain things at greater length, maybe, repeat things to him because he hasn't understood everything.” WHP10*

In order to appraise the effects of their adjustment to low HL level, HCPs said they observed patients’ health behaviors over time. They got more confident in their effectiveness if they found that after their visit, patients took their treatment better or attended more regularly medical follow-up visits.

*“I think it's really from the beginning, the first time you take the patients in charge, that you have to get into that sphere, to measure their literacy, in order to really have an impact. Afterwards, if they don't hear it from the start, they will actually come back [to the hospital]... “ WHP13*

In-hospital HCPs also emphasized that their goal was that patients get enough information and understanding to ensure their safety and self-care once returned home.

*“what is important is that at the end of the hospital stay, the patient knows about the disease, the treatment, knows what to take, how to take it, how to adapt to the treatments” MHP1*

##### Patients’ perspective

Despite the efforts described above by hospital HCPs to adapt their communication, patients reported hospital discharge as a challenging time. Some patients (4/15) said information was missing or that explanations about treatment were not sufficient.

*“I need someone to explain to me, to tell me, there you go, you've done that, the treatment corresponds to what you have, you are going to be fine. You don't just let people go out like that, without...” LWPA7*

They deplored having received information only from junior physicians, and 3/15 patients stated that they had not seen any senior physician to prepare their discharge. Conversely 2/15 said they received too much information or advice and they experienced it as a mental burden.

*“They put too much information in my head, you have to be rigorous, you have to take your treatment properly, you should absolutely not smoke, you shouldn’t use illicit products, drugs, all that, I don't use drugs. Be careful; eat a balanced diet, not too rich.”LMPA9*

Regarding information transmission between in-hospital and community doctors, most of the time, the information given to patients was consistent between hospital and general practitioners. However, 5/15 patients stated their general practitioner did not get information about their hospital stay from the hospital staff.

*“I went to see my general practitioner yesterday, he was not aware of it, he told me, I had no feedback about what happened to you. [...] I didn't get the scanner, the hospitalization; I got nothing, no information.” LWPA7*

In addition, relationships between patients and community HCPs did not always appear to be good and could constitute an additional barrier to the transmission of information.

*“She [the nurse] comes once a week. She measures my blood pressure. [...] They [nurse and GP] have no clue [about the HF follow up process]. My GP, it's the same thing. The first time, I went to see him about it, the second time, I said, it's not even worth it. He doesn't even seem to care at all. I thought they didn't know enough about it.” AMPA10*

Finally, some patients (4/15) expressed a need for more tailored information beyond the general information provided. For instance, they requested referral to other professionals depending on their needs, for follow-up by a psychologist, a tobacco specialist, or for obtaining daily help.

*“I would have liked to see someone who's accredited, a psychologist, so that I could talk about it, about what I've been through, what I've had. Well, no, I didn't receive any guidance.”LWPA7*

#### Determinants for implementing HL-tailored healthcare professional-patient interactions

##### Barriers and facilitators perceived by HCPs

Adapting one’s behavior to patients experiencing comprehension difficulties requires adaptation and training. Even though most of the hospital HCPs interviewed were considering themselves as taking the necessary time to do so, we identified four types of barriers that may influence HL-tailored communication practices: beliefs of HCPs, the place of caregivers, inappropriate documentation, and organization (lack of time, continuity of care).

HCP individual belief about patients’ competencies were directly associated with a feeling of low self-efficacy:

*“There is also the investment of the person... The fact of thinking “well, I'll get “nothing more” [from this patient] anyway” WHP3*

We also identified challenges related to informal caregivers. Some patients’ relatives took an overwhelming place in the patient-provider relationship and communication, preventing the professional from communicating directly with the patient, especially in case patients did not speak French.

*“I don't like having to go through someone else, it's a third party, it's an intermediary and uh the biggest problem is that, from time to time, I know a little bit of the language, and well the interpreter will change what I said.” MHP2*

The available documentation that HCPs may use to support their statement or to convey information to patients did not always fit the needs of patients. It may be at the same time insufficient and too detailed.

*“(we need) simple things, otherwise they don't read long blocks, some read but others tell us frankly I didn't read, or else they prefer an explanation as well.” WHP4*

Some HCPs (8/14) pointed out a significant lack of time or a lack of staff at the hospital to properly inform and educate patients before discharge. They also pointed out challenges in care continuity with a lack of post-hospital follow-up and a lack of communication between the medico-social and hospital domains that act like silos.

*“I would need an outside network that provides us with what we don't know how to do in the hospital, which is to go into patients’ homes, which is to develop relationships with nurses in private practice, which is to have a coordinator in the network, or support platforms" WHP7*

These barriers were described differently depending on the profession of HCPs. Nurses put forward the lack of communication and the informal transfer of tasks from the physician to the nurse as challenging in daily practice.

*“nurses often find themselves in the situation where they have to rephrase the diagnosis [...] and then the patient bursts into tears because they knew how to say the words and the patient understood, and actually for the patient, it’s as if it was the first announcement, and then they’re in trouble because normally it's the physician who has to make the announcement.” WHP7*

A similar vision was shared by the assistant nurse who pointed out a lack of physician interaction.

*“Because often when we go behind them [doctors] they [patients] say “they gave me the prescription but I didn't understand anything” (laughs). I think they don’t bother the doctors because they think that the doctors don’t have time but we may have more time I think. They must think like that the patients. ( … ) Because often behind the nurse re-explains the treatments.*” *WHP10*

However, a supportive interaction was reported between different professions *i.e. *between residents and assistant nurses for communication with patients.

*“ they [assistant nurses] talk to each other to see how they can make things easier. I’ve had quite a few assistant nurses who have helped me explain to patients what their pathology is, what treatment we’re going to give them, and everything.” WHP11*

Other facilitators that HCPs reported were related to the patients’ side and how they are engaged in their care.

*“If the patient has, let’s say, understood, and finally he/she becomes an actor ... necessarily he/she will take better care of himself/herself but ... it's like for everything else, one must understand why do things … ” MHP8*

Facilitators could also relate to community care, which was considered as more individually adapted to patients’ needs. For instance, interviewees declared that home care nurses and community pharmacists might take more time with the patient, in particular to explain the prescriptions.

*“ … the second help that we will be able to have outside the hospital it will be the pharmacist, the one who will deliver the medicines to the patients who will show him the boxes, who will show him the tablets who will tell him how he must take them....”MHP1*

##### Barriers and facilitators perceived by patients

The main barriers to a satisfying hospital discharge for patients were the lack of follow-up and continuity of care at home. Some patients reported a need for support once they returned home (6/14), while others did not declare any specific needs or issues. However, problems related to information and communication were reported. Some patients found it difficult to ask questions to their physician because of fear of judgment, or fear of disturbing (7/14).

*“Sometimes, I don't tell all my problems. I don't dare.” LWPA13*

Patients also reported a lack of opportunity to clarify and deal with the information they received in depth.

*“I had a very important question to ask them, they inserted me a stent and I wanted to know if the rest of the arteries were damaged or not. That's something I don't know anything about. So I would have liked to know if the rest of the arteries were okay. If I've got one that's got a stent and I've got one next to it that's damaged, it would be nice to know.” LMPA2*

Patients reported the fact that nurses and assistant nurses were more present and engaging ininteractions than physicians during the hospitalization period. Patients were sometimes dissatisfied with not knowing the different roles and profession of the care team members.

*“When I was there, I saw mostly residents and people, and by the way I got angry because I didn't have any communication about what was going on ... I knew what I wanted to know when I saw the physician between two doors, he talked to me in the hallway.” AMPA6*

Thus, nurses and assistant nurses were a source of support for patients, in addition to informal caregivers (family, relatives) who were identified by patients as being their main social support.

The available discharge programs, although very scarce, were appreciated by the few interviewed patients who were included in such programs. For instance, “PRADO” (the program developed by the French public health insurance system that allows patients to be monitored at home for 2 months after hospitalization) was reported to provide more regular follow-up and social support.

*“At least I'm seeing someone, that's good. She measures my blood pressure, she weighs me...” AMPA6*

Contrary to the sometimes overwhelming presence of caregivers mentioned by HCPs, patients perceived them differently as very helpful. Patients used material support tools to improve their self-management capabilities, such as pillboxes to organize their treatments, and caregivers or community nurses often managed these for patients who needed it.

*“ They are the ones who manage the pillbox, they are the ones who manage everything” (talking about the nurses) Daughter of LWPA1*

## Discussion

Overall, the findings of the present study highlighted the gap between the perceptions of patients and HCPs regarding the adaptation of practices to the HL level at each step of the patient care pathway. This qualitative study provided insights into the perspectives of patients and HCPs regarding the barriers and facilitators to implement HL-tailored communication for the management of CVD. We proposed individual, interactional, and organizational strategies that may be effective in improving the uptake of evidence-based HL-tailored practices, and the access to care and health outcomes for low-HL patients (Fig. 1). These results will help in the development of an intervention planned within a broader project aiming at reducing the gap between HL knowledge and practices as discussed below.

### Individual factors

Even though HCPs expressed their intention to tailor their communication according to patient difficulties, the complete definition of HL was not clear to them, and they expressed a lack of tools [36] to identify low-HL patients and act accordingly. Additionally, they acknowledged the need for tailored communication in the way of communicating with such patients. Hence, providing them with information to act up on their perception in order to adapt their own behavior and practices of communication would be an appropriate solution to improve patient understanding. Thus, their beliefs often called upon self-attribution regarding interaction with patients, as shown by Nache & Trudeau *et al.* [37]. When this strategy failed, they more easily referred to external allocation, putting forward the lack of means and lack of time they face in their daily practice. We also identified that HCPs who were more in regularly contact with patients (nurses, care assistants) were more likely to report delivering HL-tailored support, which is in line with published studies advocating that addressing HL should be a nursing skill [38]. Still, regardless of their profession, many HCPs lacked knowledge, skills, and practical tools to routinely and appropriately adopt HL-tailored practices in hospital care [39].

### Interaction factors

Interactions between patients and HCPs constitute a challenging issue to overcome. Indeed, most HCPs rely on patient commitment to express their needs, while low-HL patients can either hide their misunderstanding behind the existing communication difficulties or fear the stigma attached to them [40]. HCPs mainly gave partial definition of HL, ignoring all the dimensions of HL described by Nutbeam *et al*. [36] that constitute a qualitative interaction [41]. The surplus of information provided at the time of hospital discharge and the need for specific information once back at home showed that HCPs may overestimate patients’ understanding of the post-discharge plan [42]. Upgrading methods for effective information transmission has been shown to improve outcomes in terms of disease knowledge, adherence, self-efficacy, and also to reduce the hospital readmission rates for chronic disease [3, 10, 43].

Moreover, both patients and HCPs pointed out a gap in the transition between hospital and community care. In community care, low-HL patients’ difficulties to communicate with HCPs may persist with their general practitioner (GP). Communication with the GP regarding hospital stay was occasionally reported as limited. This point has to be improved since the transmission of hospital stay information cannot only rely on patients’ ability to summarize their situation and represents an unnecessary responsibility for patients. A quality improvement study has demonstrated how educational sessions can improve discharge summaries and communication with primary care [44].

### Organizational factors

Regarding organizational challenges at the hospital, both patients and HCPs underlined staff shortages, which may limit interactions between senior physicians and patients. A lack of patient empathy and confidence towards residents in patient centered care and communication has also been reported [45]. This illustrates that a long-term patient-HCP relationship of trust needs to be built and maintained, especially in the context of chronic diseases [46]. At the hospital, HCPs support each other and are complementary when dealing with patients’ difficulties. This may as well allow the distribution of skills and tasks between each profile. Di Palo *et al.* have showed a good coordination in the management of HF patients improved health outcomes [47].

As hospital discharge constitutes such a defining step for patient care, in addition to the strategies discussed above, further solutions need to be implemented to establish a link between hospital and community care. Once again, on a relational level, other key players such as the pairing of the nurse/care assistant or the community pharmacist may contribute to a successful discharge [48]. Additionally, in terms of organizational resources, time needs to be allocated to this essential moment. Moreover, transition at the individual level may be improved by social support [49], for instance by mobilizing family, or proposing instrumental aids such as pillboxes.

### Implementation strategies

**Fig. 2:**
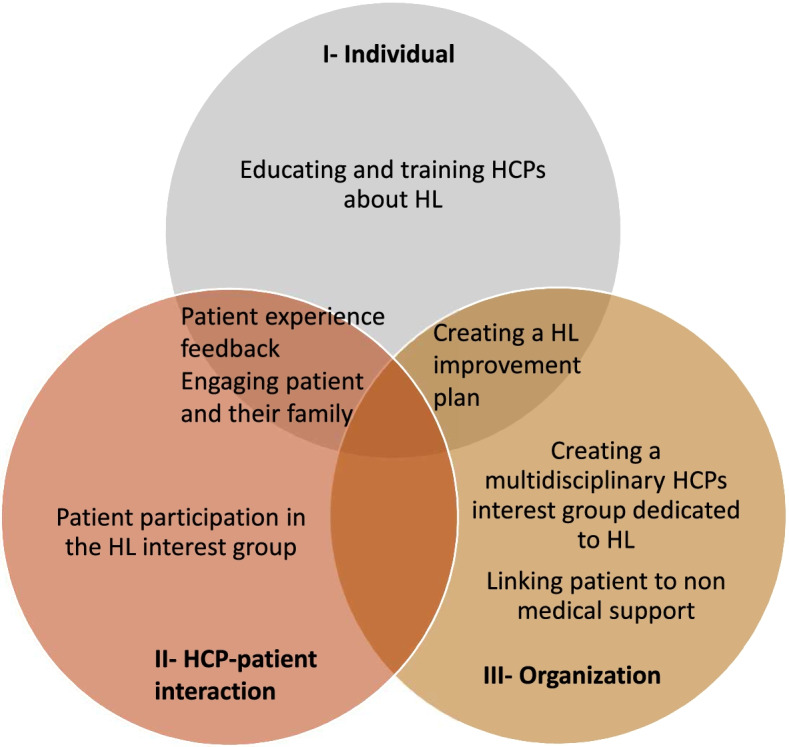
Diagram linking the three groups of Ishikawa barriers and facilitators with the proposed implementation strategies

Considering these results, we identified several implementation strategies that might drive the translation into practice of evidence-based HL-tailored communication strategies [43], improve patient-HCP interactions, and create a culture of HL in the management of CVD [34, 35]. We summarized in Fig. 2 implementation strategies that might be relevant in the discharge planning of patients hospitalized for HF according to our results. First, *educating and training HCPs about HL* is a prerequisite. Nowadays, in France, HL and its related issues are not part of neither the regular medical education curriculum nor continuous education. Teaching HL within medical education should ensure all HCPs share the same core knowledge on HL, better understand the challenges related to low HL level, and develop skills to identify and communicate with low-HL patients.

Second, *creating a multidisciplinary HCP interest group dedicated to HL* might promote best practices for low-HL patients and induce peer-to-peer exchange at the organizational level [34, 35]. The multidisciplinary dimension of such group would enable to reflect on the role of all HCP profiles (physicians, nurses, care assistants, students). The tasks and activities of this group may be to provide continuous training/education sessions on HL in the department, to develop HL toolkits specific for each profile of HCPs, and to provide evidence-based HL communication methods. Finally, the interest group may act as a local champion and facilitator to develop a HL culture and drive changes by *creating a HL improvement plan* constituted of short and long-term goals for addressing HL challenges in the context of CVD care.

Third*, engaging patients and their family* would also encourage HCPs to move evidence into practice [34, 35]. This may be done by combining different approaches such as *patient participation in the HL-interest group*, patient involvement in designing the *HL plan*, and setting-up a systematic *patient experience feedback process* (patient experience survey for instance). This could enable HCPs to identify patients’ needs and priorities and confirm communication improvements and successes with low-HL patients [50].

Fourth, regarding the gap perceived by patients between hospital and ambulatory community setting, involving GPs and pharmacists alongside patients and families may help to design innovative solutions to share information, or introduce new professions such as coordinating nurse [47] or case manager for chronic diseases [51]. L*inking patients to additional non-medical support,* such as a community-based network or support by expert-patients may also help address patients’ issues during care transitions.

Our study has some limitations. First, the interviewed patients were not followed directly by the HCPs interviewed, hence we cannot directly link the two perspectives. This may have hindered the possibility to explore whether individual strategies of HCPs were effective from their patients’ perspectives, and identify how HCPs directly adapt to specific patient situations. However, structural determinants, such as the location of the interview or the department from which the HCPs originated did not influenced the results. We may hypothesize that HCPs share the same education, training and sensitization to HL. Indeed, all interviewed patients were hospitalized in a unit where interviewed HCPs practiced, and all HCPs worked with patients with HF and MU, and were therefore able to answer our questions. This also allowed each of the participants to be neutral and free to answer as they wished. Moreover, interviewing HCPs and their patients to compare their views would have biased discourses towards an evaluation or satisfaction interview, and induce a social desirability bias. Second, for patients with communication or interactive HL difficulties, the expression of their issues may have been hindered by the fact that the interviews were not conducted in a neutral place but at the hospital. However, this risk was reduced by the attitude and profession of the interviewer (AP), who is not a HCP. The hospital where patients were recruited is a large hospital that covers a large population and geographical area, so it was not possible for us to go to the patients’ home, and a place different from the hospital while attending an encounter was not convenient for patients. However, patients were interviewed in the outpatient clinical and not in the inpatient ward; this may have reduced the psychological impact and the influence of the place where the interviews took place on patient discourse. Outpatient clinic is more neutral since the interviews targeted the hospital stay, hospital discharge, and return home.

The HCPs interviewed were volunteers and made themselves available to participate in the study, which probably biased towards the inclusion of the participants who were more aware of the concept of HL and interested in the topic, especially since some were recruited through colleagues or superiors involved in health education approaches that were closely related to HL. However, for feasibility reasons, it was not possible to adopt other sampling strategies of recruitment, and circumventing the volunteer bias is very challenging especially for qualitative research. However, our results showed that, despite this potential bias, HCPs’ knowledge regarding HL remains partial, illustrating that it is essential to implement interventions to promote good communication practices.

Finally, we are aware that the tool chosen to measure patient HL does not reflect all aspects of HL and remains a subjective tool. However, we needed this tool to be administered to all patients in the quantitative P-ILIADE study to categorize patients and identify the barriers specific to the patients presenting more difficulty in the present qualitative portion of the study.

## Conclusions

The exploration of the perceptions of HCPs and patients regarding their interactions at discharge showed both commonalities and differences regarding the care pathway. Our study provides useful information on which implementation techniques at individual (educating and training of HCPs about HL), interactional (patient participation in the HL interest group), and organizational level (creating a multidisciplinary HCP interest group dedicated to HL) can be built. These may be effective to improve communication with low-HL CVD patients in order to improve discharge planning. Involving hospital and community HCPs, patients, and family members within a co-construction strategy will be one of our future goals to operationalize our results in the development and implementation of the CVD management intervention regarding discharge.

## Supplementary Information

**Additional file 1.**

## Data Availability

Verbatim from this research are available upon reasonable request to the corresponding author.”

## Abbreviations

HCPs: Healthcare professionals
HL: Health Literacy
CVD: Cardiovascular Disease
MI: Myocardial Infarction
HF: Heart Failure
TBP: Theory of Planned Behavior
HLHA: Health Literacy and Health Action
BHLS: Brief Health Literacy Screening questionnaire
GP: General Practitioner
