# Supplementary material for: Considering health literacy in cardiovascular disease management: a qualitative study on healthcare professionals’ and patients’ perspectives
Source: BMC Health Serv Res. 2022 Sep 5;22:1121. doi: 10.1186/s12913-022-08455-8 (PMC9446730; doi:10.1186/s12913-022-08455-8)
Supplement: Supplementary file 1 — Additional file 1. [file 12913_2022_8455_MOESM1_ESM.docx]

**Appendix 1.** Overall final model for analysis

**
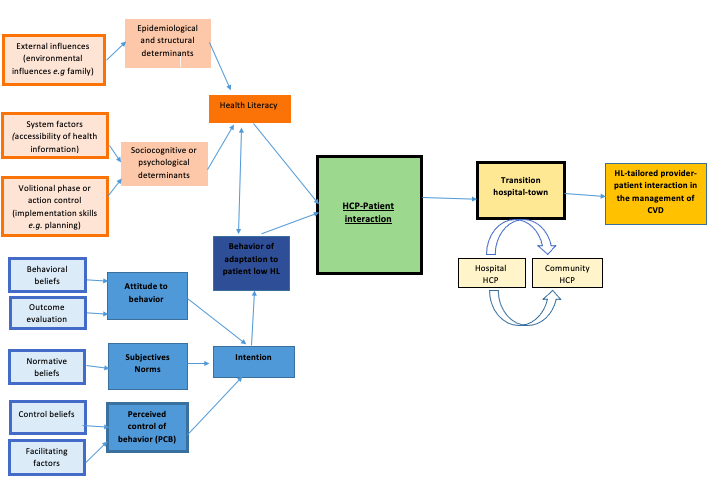
**

**Appendix 2: Interviews guides**

Table A: Topics covered during patient interviews

***AIM: Evaluate the information needs of patients regarding their pathology and its management one month after hospital discharge.***

| **THEMES** | **TOPICS** |
| --- | --- |
| **Motivation to stay healthy** | -The self-management of the patient’s disease.  -Feelings after hospital discharge.  -Changes of habit related to the disease  -The projects in life, the dreams… |
| **Knowledge of the disease** | -Patients’ understanding of their disease  -Importance of disease management  -Analysis of warning signs. |
| **Difficulties in managing the disease on a daily basis** | -Knowledge, competency, motivation about treatment, hygienic and dietetic rules, medical monitoring, etc.  -The management of treatment.  -Lifestyle change(s) after hospital discharge.  -The medical follow-up in the month following hospital discharge. |
| **Experiential feedback about the month following hospital discharge.** | -The information provided at hospital discharge (documents…)  -The needs of the patient after hospital discharge and once back home (difficulties, positive things).  -Things that could have helped them. |
| **Interaction with health care professionals** | -Patient’s observations and experiences of the relationship.  -The most suitable professional to help in managing one's pathology.  -Ideas for better communication with professionals. |
| **Health information** | -How the patient accesses information (difference between hospital and community care).  -Information needs to help manage one's pathology.  -Ideas for help in finding the right information. |
| **Self-efficacy** | -How the patient perceives its effectiveness related to his or her health.  -The difficulties in terms of ability to manage effectively their disease.  -Assessment of family support.  -The thing that would help them the most. |

***Main questions regarding themes and topics presented above and interview introduction:***

Hello. Do you remember we talked about this a month ago? At the time of your hospitalization, you agreed to participate in a discussion, as part of the health literacy study also known as health literacy skills.

So, following your hospitalization for a myocardial infarction/heart decompensation episode last month, we would like to know how your return home went.

Are you still willing to participate in this interview?

In order to transcribe your words as well as possible and to facilitate the analysis, I will record the interview. But rest assured, this is completely anonymous. Everything you say will be used solely for research purposes and none of the information will be shared with the doctors or medical team.

Emphasis:

*Non-medical > No information will be given to the medical team here or in town.

*The main thing is to discuss and understand your experience in order to improve the care you receive

*The idea is to listen to you and have you share your experience of what it was like to go home.

*Any anecdote or experience is interesting and I am not here to judge you, you will contribute to change

**Motivation to stay healthy**

How do you feel after your hospitalization?

What are you trying to do to get better and feel healthy?

Do you have any plans for your life, any dreams?

**Knowledge of the pathology**

What is most important to you in managing your heart condition?

*What do you understand about your condition?

Do you know how to identify symptoms/warning signs related to your condition?

*If so, do you know where to turn if you recognize these warning signs?

**Difficulties in managing the disease on a daily basis regarding**

**o Medication**

Did you take your medication after discharge from the hospital?

How many medications do you need to take every day?

Do you know what treatments you have to take?

Do you have any barriers to taking your medications?

Do you know who to go to/consult with if you have questions about the treatments you are taking?

Can you manage your DTC on your own (without help from a third party)?

**o Diet and health rules**

Did you change your way of life after this hospitalization?

**o Medical follow-up**

Did you have any medical follow-up (nurse, attending physician, specialist, other medical professional, PRADO) in the month following your hospitalization?

**Experiential feedback about the month following hospital discharge.**

When you were discharged from hospital, what information were you given?

Once back home, what were your needs, related to this hospitalization and the information you received to manage your pathology?

What could have been put in place / Things that could have helped your return home?

*More or less information?

*Clearer information?

Do you have caregivers (family, friends, etc.) who help you manage your condition?

**Interaction with health care professionals**

Which health professional(s) support(s) you in the management of your disease (nurse, physiotherapist, pharmacy, doctor, etc.)?

What could be put in place to better communicate with the professionals?

**Health information**

What do you need in terms of information to help you manage your health differently?

What would help you find good information about your health and what is best for you?

Was the information different between what was given in the city and in the hospital?

**Self-efficacy**

Do you have difficulty managing health concerns in general?

Do you feel you can manage your health as well as anyone else?

Of all the things, we have talked about now, what would help you the most?

On a scale of 1 to 10, how would you rate the help of your family/caregiver?

1 patient alone 5 caregiver = patient 10 caregiver

Table B: Topics covered during HCPs interviews.

***AIM: Assess health care professionals' perceptions of patient health literacy levels and identify communication modalities based on health literacy.***

| **THEMES** | **TOPIC** |
| --- | --- |
| **Health literacy *representation*** | -Understand where knowledge comes from.  -Motivation.  -Experience around health literacy |
| **Definition of patients' health literacy** | -How professionals observe and understand patient’s low HL.  -Observation of patient’s difficulties.  -Factors and determinants for identifying low-HL patients. |
| ***Behavior* of the health professional when dealing with a patient with low health literacy skills** | -Adaptive professional behavior regarding interactions and communication with low-HL patients.  -How they care for a patient with low HL (word used, discourse).  -The role and position in the relationship. *(perceived control).*  -The barriers (organizational, human, materiel) *(barriers).*  -The influence of the family.  -The influence on the hospital discharge. |
| ***Attitude to behavior*** | -Beliefs about the consequences associated with the behavior.  -Assessment of the value of consequences.  -The effects of caring for a low-HL patient.  -Professionals' observations and experiences.  -Adaptation of practice according to the number of hospitalizations/visits (monitoring of illness / treatment / lifestyle changes / adherence to medication).  -The personal beliefs of the adaptation in his/her practice. |
| ***Social identity*** | -Professionals' observations and experiences.  -The communication with his/her colleagues in particular regarding the transmission of information.  *-Subjective norms* |
| **Barriers and facilitators for low HL**  ***(perception of control over behavior)*** | -Professionals' observations and experiences.  -The tools (and ideas) to better respond to the patient's discharge from hospital.  -The tools they have to help the patient understand his or her pathology.  -Needs of professionals. |

Abbreviations: HCPs: health care professionals; HL: health literacy.

***Main questions regarding themes and topics presented above and interview introduction:***

"Hello, my name is ............. and I am a student in ......... in a public health research laboratory (xxxx).

I am currently interested in the topic of health literacy and cardiovascular pathologies. The two pathologies of interest are heart failure and myocardial infarction. I am currently conducting a study on this subject in order to better understand the issues involved in the management of these patients. Thank you for agreeing to conduct an interview on this subject.

I will ask you some questions and the goal is to understand a little better your relationship with the patient and in particular, the associated challenges.

This interview will be recorded in order to transcribe your words as well as possible, but it will be completely confidential and used exclusively for the purpose of the research.

There are no right or wrong answers, it is your word that is important.

Do you have any questions before we begin?"

How do you picture the concept of health literacy?

How do you identify low health literacy in your patients?

Can you describe how you manage a patient with low health literacy?

What do you think is most important in managing a patient with low health literacy who is hospitalized for one of the two conditions of interest? (To ensure a successful discharge from the hospital)

Based on the frequency of visits/hospitalizations of a patient with low health literacy, how do you adapt your practice?

Do you communicate information about the low health literacy patient to your peers or to the health care team?

With the study goal of building an educational tool to meet the needs of patients, what might be a tool to better address their challenges at discharge?

***Appendix 3.*** Key findings from patients and HCPs interviews following the overall model analysis (adjacent theoretical constructs in *italics*)

|  | **I-   HCPs’ knowledge on health literacy** | **II-  HCP-patient interactions: current practices perceived by HCPs and patients** | **III-  Determinants for implementing HL-tailored HCP-patient interaction** |
| --- | --- | --- | --- |
| **Healthcare professionals** | 1. **Low knowledge, no or partial definition (**functional HL) 2. Low-HL patients represented by their educational level, social features, or behavior   *(normative belief)* | 1. Adapting the way of delivering information (*i.e.* taking the time, illustrate)   (*perceived control of behavior/facilitating factors)*   1. Relying on the patient's commitment, 2. Aiming to make him/her an actor and his/her safety once discharged   (*outcomes evaluation)* | 1. **Barriers:** Human, organizational, material barriers   (*control belief, normative beliefs)*   1. **Facilitators**: inter-professional support, patient engagement, link with community HCPs   *(facilitating factors)* |
| **Patients** | / | 1. Either **insufficient** and **overwhelming information** losing the patient and increasing the mental burden 2. Need for **better transmission of information** from hospital to general practitioner 3. **More specific information** from appropriate HCPs such as psychologist or tobacco specialist   (*system factors)* | 1. **Barriers:** lack of follow-up and listening (home or hospital)   *(system factors)*   1. More interaction with nurses and assistant nurses than physicians   *(patient-provider relationship)*   1. **Facilitators**: caregivers (social support)   *(external influences)*   1. Organizational and follow-up tips for taking ownership of information   *(volitional phase/action control)* |

**Abbreviations:** HCPs: healthcare professionals, HL: health literacy.
